# Association between Lipids, Apolipoproteins and Telomere Length: A Mendelian Randomization Study

**DOI:** 10.3390/nu15214497

**Published:** 2023-10-24

**Authors:** Gehua Zhu, Jiamin Xu, Guanghua Guo, Feng Zhu

**Affiliations:** 1 Medical Center of Burn Plastic and Wound Repair, The First Affiliated Hospital of Nanchang University, Nanchang 330000, China; zhugh2023@163.com (G.Z.); xujiamin_1020@163.com (J.X.); 2 Department of Critical Care Medicine, Shanghai East Hospital, Tongji University School of Medicine, Shanghai 200120, China

**Keywords:** lipids, apolipoproteins, telomere length, mendelian randomization

## Abstract

(1) Background: The relationship between lipids, apolipoproteins, and telomere length (TL) has been explored in previous studies; however, the causal relationship between the two remains unclear. This study aims to assess the causal relationship between lipids, apolipoproteins, and TL using the two-sample Mendelian randomization (MR) approach; (2) Methods: This study comprehensively employed both univariate MR (uvMR) and multivariate MR (mvMR) methods to genetically evaluate the associations between 21 exposures related to lipids and apolipoproteins and the outcome of TL. During the analysis process, we utilized various statistical methods, including Inverse Variance Weighting (IVW), Weighted Median, MR-Egger regression, MR-PRESSO, and outlier tests. Furthermore, to confirm the robustness of the results, we conducted several sensitivity analyses to explore potential heterogeneity; (3) Results: The uvMR analysis indicated that an increase in MUFA, MUFA/FA ratio, LDL-C, VLDL-C, total cholesterol, ApoB, and triglycerides (TG) was associated with an increase in TL. However, this relationship did not manifest in the mvMR analysis, suggesting that this association may be based on preliminary evidence; (4) Conclusions: MR analysis results suggest potential suggestive positive causal relationships between genetically predicted MUFA, MUFA/FA ratio, LDL-C, VLDL-C, total cholesterol, ApoB, and TG with TL.

## 1. Introduction

Telomeres are specialized nuclear protein structures closely associated with age-related diseases, making them a potential biological marker of aging. Human telomeres consist of highly conserved hexanucleotide repeat sequences (TTAGGG) rich in G at the ends of eukaryotic chromosomes and associated proteins, forming a protective cap at the chromosome ends called the T-loop. This structure remains inactivated in response to DNA damage pathways or chromosome fusion events. However, due to the inability of the cell replication machinery to fully replicate the chromosome ends, 50–100 base pairs are lost with each cell division, resulting in the gradual erosion of telomeres. Consequently, as cells age, TL diminishes.

Telomeres are essential for maintaining genomic stability at the ends of chromosomes. As cells divide and DNA replicates, telomeres gradually shorten due to the “end replication problem.” To some extent, TL is heritable and influenced by factors such as gender, race, and paternal age. Factors negatively correlated with TL include prenatal [1] and childhood [2] stress, chronic stress in adult life [3], as well as conditions like depression [4], smoking [5], obesity [6], and alcohol consumption [7], all of which accelerate telomere shortening. Telomere shortening can be prevented by dietary restriction [8] and increased intake of dietary antioxidants [9]. Dietary intake is a significant determinant of cellular TL.

TL is primarily regulated by telomerase. Telomerase is a ribonucleoprotein that can compensate for telomeric loss incurred during cellular division—a complex consisting of catalytic subunit reverse transcriptase (TERT) and RNA component (TERC). At the same time, it is also subject to modulation by specific proteins, such as WRAP53. In a cross-sectional analysis, researchers examined the relationship between lipoprotein subfractions and TL and the expression of TERT and WRAP52 in 54 prediabetic individuals from the EPIRDEM study. The findings revealed a positive correlation between smaller-sized high-density lipoprotein (HDL) particles and shorter telomeres, along with lower TERT and WRAP53 expression levels. Conversely, larger-sized HDL particles were positively associated with longer TL, although unrelated to TERT. Hence, the study concluded the existence of a correlation between the lipoprotein profile and TL, as well as the expression of TERT and WRAP53 [10].

Prior metabolomics research has unequivocally indicated that lipid metabolism plays a pivotal role in regulating TL. Various metabolites derived from fatty acids, such as glycerophosphocholine, glycerophosphoethanola-mine, lysophospholipids, glycerides, and phosphatidylcholine, are closely associated with TL [11]. Additionally, lipoproteins, particularly HDL-C, along with total cholesterol and TG, have been consistently found to be linked to TL in multiple studies [12,13]. These findings underscore the intricate relationship between lipid metabolism and TL, providing a robust foundation for further exploration in this field.

A study revealed a positive correlation between TL and polyunsaturated fatty acids (PUFAs), including linoleic acid, in 174 healthy adults [14]. Another analysis of data from 11,775 individuals across six independent population cohorts found positive associations between total cholesterol in small VLDL and the total lipid ratio, total cholesterol in small VLDL and the total lipid ratio, the ratio of w-6 fatty acids to total fatty acids, and the ratio of 18:2 linoleic acid to total fatty acids with TL [15]. However, these relationships have not been conclusively confirmed.

Within the PUFA category, *n*-3/6 PUFAs are two major families closely related to human health [16,17]. In the *n*-3 PUFAs family, alpha-linolenic acid (ALA) is considered an essential fatty acid. In healthy young males, approximately 8% of dietary ALA is converted to eicosapentaenoic acid (EPA), and up to 4% is converted to docosahexaenoic acid (DHA). Long-chain fatty acids from marine sources, such as EPA and DHA, have shown significant benefits in maintaining balance and preventing diseases, receiving extensive research attention. It is important to note that *n*-3 PUFAs serve not only as an energy source but also as major biological factors in normal growth, development, and disease regulation.

Animal studies suggest that feeding rats with diets rich in *n*-3 PUFAs can slow down telomere attrition and extend telomeres [18]. Another study indicated that supplementing with *n*-3 PUFAs could improve liver TL in offspring of mothers with gestational diabetes [19]. However, there is still controversy regarding the benefits of *n*-3 PUFAs in humans. One study found a significant association between a higher *n*-6/*n*-3 PUFAs ratio and shorter TL. Although this association was related to increased *n*-3 PUFAs, it appears that *n*-3 PUFAs have no actual impact on telomeres [20]. Moreover, epidemiological studies have shown no significant association between the combined levels of ALA, EPA, and DHA in red blood cells and leukocyte TL [21].

Hence, it is crucial to gain deeper insights into the causal relationships between lipids, apolipoproteins, and TL. MR, as an emerging epidemiological method, assesses causal relationships between exposures and outcomes by utilizing genetic variants as instrumental variables. MR’s advantages lie in minimizing the influence of confounding factors, thereby greatly reducing interference from confounding variables between exposure and outcome [22,23]. To investigate the potential causal relationship between PUFAs and TL, we conducted MR analyses using summary-level data from genome-wide association studies (GWAS) on two samples and validated the findings using other datasets.

## 2. Methods

### 2.1. Study Design

**Figure 1:**
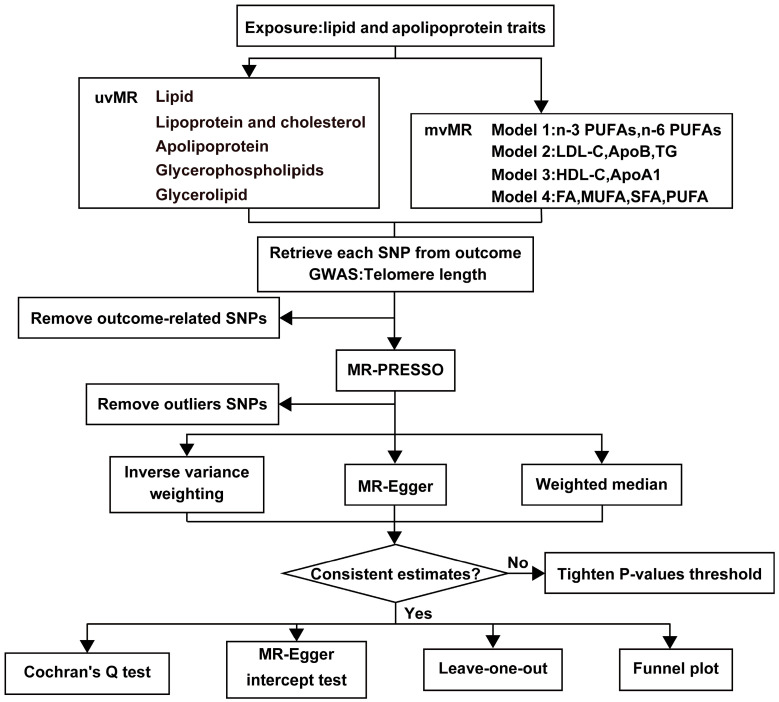
The workflow of MR analysis. HDL-C = HDL cholesterol, LDL-C = LDL cholesterol, ApoA1 = Apolipoprotein A1, ApoB = Apolipoprotein B, TG = Total triglycerides, FA = Total fatty acids, MUFA = Monounsaturated fatty acids, SFA = Saturated fatty acids, PUFA = Polyunsaturated fatty acids.

Our study is based on three fundamental assumptions, similar to most MR analyses. These assumptions are as follows: (1) There is a strong correlation between genetic variants and exposures; (2) Genetic variants are unrelated to the exposure-outcome association and are not influenced by confounding factors; (3) Genetic variants exert their effects solely through the association between exposure and outcome [24]. Figure 1 provides an overview of our study design. We utilized de-identified data openly available from participant studies, which have received ethical committee approval for human experimentation. This study did not require separate ethical approval. The reporting of the study follows the requirements of the STROBE-MR guidelines.

**Figure 2:**
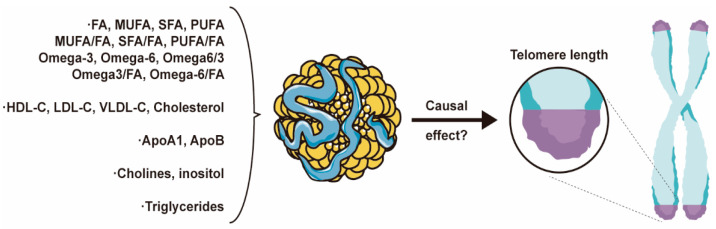
Core Figure of the Study: Lipids and Apolipoproteins Impact Telomere Length.

This study categorizes the exposure factors into five major groups, as follows: (1) Lipid categories: encompassing total fatty acids (FA), monounsaturated fatty acids (MUFA), saturated fatty acids (SFA), PUFA, *n*-3 PUFAs, *n*-6 PUFAs, and their respective ratios; (2) Lipoprotein and cholesterol categories: comprising HDL-C, low-density lipoprotein cholesterol (LDL-C), very-low-density lipoprotein cholesterol (VLDL-C), and total cholesterol; (3) Apolipoprotein categories: including apolipoprotein A1 (ApoA1) and apolipoprotein B (ApoB); (4) Glycerophospholipids categories: encompassing choline and myo-inositol; (5) Glycerolipid categories involving TG (Figure 2). We employed two distinct MR analysis methods. First, we conducted univariate MR analyses for each of the 21 exposure factors individually to investigate their independent effects on the outcome. Subsequently, we constructed four models and applied the mvMR analysis method to examine the relationships between the exposure factors.

### 2.2. Lipid and Apolipoprotein Data Sources and Instrumental Variables

Regarding the lipid and apolipoprotein data used in this study, GWAS summary data were sourced from the UK Biobank for all exposures except myo-inositol, which was obtained from human blood metabolites analyzed by Shin et al. in 2014 [25], covering 7803 samples, as detailed in the supplementary table. We first selected genome-wide significant single nucleotide polymorphisms (SNP) (*p* < 5 × 10^−8^) from the GWAS and excluded SNPs with low linkage disequilibrium (R^2^ < 0.001). Additionally, we utilized the PhenoScanner V2 database, which provides comprehensive genotype and phenotype association information, to assess and exclude SNPs related to other phenotypes, including potential confounders and intermediate variables. When considering the concordance of PUFA with the outcome, we also removed SNPs with palindromic or incompatible alleles. The strength of each Instrumental Variable (IV) was assessed using the F-statistic, with an F-statistic below 10 considered a weak IV [26].

### 2.3. Outcome Data

Genetic variants associated with TL were extracted from the largest GWAS dataset to date, obtained from the UK Biobank [27] (Table S1). This dataset represents a massive cohort study analyzing 20,134,421 SNPs and includes 472,174 individuals aged between 40 and 69 years. The outcome dataset has undergone statistical adjustment for age, removing the influence of age on TL. Among the participants, 45.8% were male, and 54.2% were female, with a balanced gender ratio. The dataset’s racial composition is primarily European Caucasian, with 94.3% being white, 1.9% Asian, 1.5% Black, 0.3% Chinese, 0.6% mixed race, and 0.9% other ethnicities. DNA was extracted from peripheral blood leukocytes in the UK Biobank cohort, and TL was measured as the T/S ratio using quantitative polymerase chain reaction methods.

### 2.4. Outcome Data

#### 2.4.1. Univariate MR Analysis

We employed various methods for testing, including IVW, weighted median, MR-Egger regression, and MR-PRESSO. The IVW method served as the primary statistical approach to estimate potential causal relationships between lipids, apolipoproteins, and TL. To assess the significance of heterogeneity at the multivariable level, we first used Cochran’s Q-test to evaluate heterogeneity, with Cochran’s Q yielding a *p*-value less than 0.05 indicating IV heterogeneity. Subsequently, we further assessed the absence of horizontal pleiotropy using MR-Egger intercept tests and exclusion analysis, with a *p*-value greater than 0.05 suggesting no horizontal pleiotropy. Additionally, we employed the MR-PRESSO method to detect and correct for horizontal pleiotropy outliers in all reported results from the multivariable summary-level MR tests [28]. After excluding outlier SNPs, we conducted robust MR calculations. Finally, funnel plots were used to assess potential directional pleiotropy, and leave-one-out analysis evaluated whether the association was driven by individual SNPs [29].

#### 2.4.2. Multivariate MR Analysis

To account for pleiotropy across lipid traits, we conducted mvMR analysis, constructing four models primarily using the multivariable IVW method. Model 1 included *n*-3 PUFAs and *n*-6 PUFAs, as these fatty acids share a relationship in structure and function, and both belong to the PUFA category. Model 2 included LDL-C, ApoB, and TG, as ApoB forms particles when encapsulating LDL-C and TG [30,31]. Model 3 included HDL-C and ApoA1, as ApoA-I is the major structural and functional protein of HDL, constituting 60% of total protein. Model 4 included FA, MUFA, SFA, and PUFA. Similar to the univariate MR, we employed Cochran’s Q-test and MR-Egger intercept tests to detect heterogeneity and pleiotropy.

**Figure 3:**
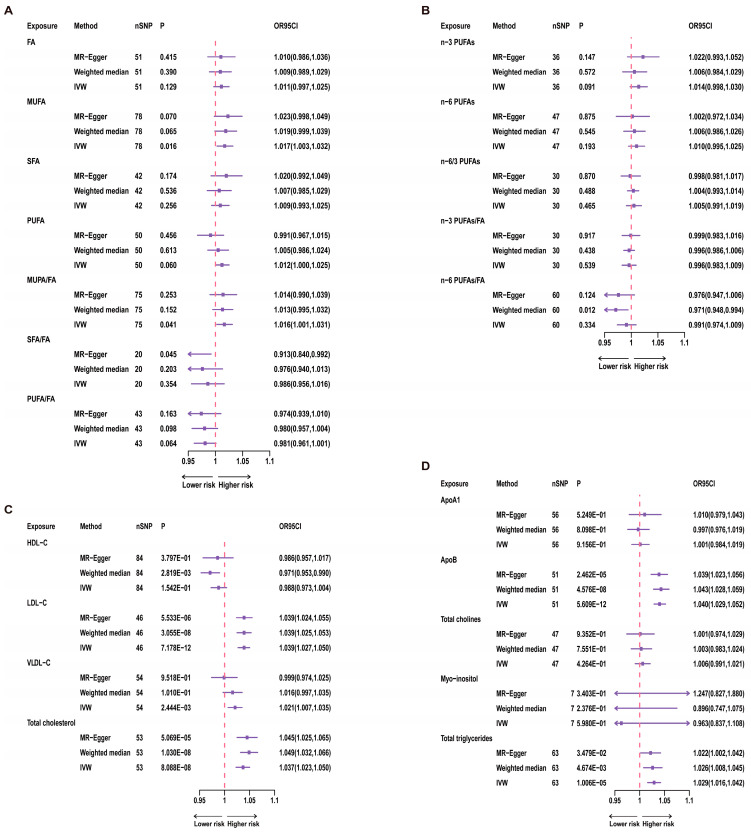
Results of univariate MR for 21 Exposures and Telomere Length. A red dashed line in the figure represents the odds ratio (OR) value of 1. nSNP = number of SNP. (**A**) uvMR results for fatty acids and TL; (**B**) uvMR results of *n*-3 PUFAs and *n*-6 PUFAs and TL; (**C**) uvMR results for lipoprotein cholesterol and TL; (**D**) uvMR results for glycerophospholipids, glycerolipids and TL.

All MR analyses were conducted in the R software (version 4.3.0) and analyzed using the R package “TwoSampleMR” (version 0.5.6). The forest plot in Figure 3 was created using R software.

## 3. Results

### 3.1. Instrumental Variables

IVs used in this study for unMR are detailed in Table S2. We also conducted a strength analysis of these instrumental variables, and the results indicated that all SNPs had F-statistics greater than 10 (Table S2), suggesting strong predictive power of our instrumental variables.

### 3.2. Univariate MR Analysis Results

#### 3.2.1. Fatty Acids

For univariate analysis of the 12 categories of fatty acids, we conducted IVW analysis. The results showed a significant correlation between MUFA and TL (*p* = 0.016, Figure 3). Specifically, for each increase of one standard deviation in MUFA, the odds ratio (OR) for TL was 1.017 (95% CI: 1.003–1.032). However, after Bonferroni correction, this result did not reach statistical significance, suggesting a suggestive causal relationship. Furthermore, the ratio of MUFA to total fatty acids also showed a significant correlation with TL (*p* = 0.041, Figure 3), while total fatty acids were not significantly correlated with TL (*p* = 0.129, Figure 3). Therefore, this relationship with TL may be primarily driven by the MUFA variable. Even after Bonferroni correction, the *p*-value for the MUFA/FA ratio did not reach statistical significance, indicating only a suggestive causal relationship. In contrast, *n*-3 PUFAs, *n*-6 PUFAs, and other variables showed no significant correlations with TL (Figure 3).

#### 3.2.2. Lipoproteins and Cholesterol

In investigating the associations between three lipoproteins and cholesterol and TL, we used the IVW analysis method. The results demonstrated a significant correlation between LDL-C (*p* = 7.178 × 10^−12^, Figure 3) and TL. Additionally, VLDL-C (*p* = 2.444 × 10^−3^, Figure 3) showed a significant correlation with TL. Moreover, the association between total cholesterol and TL also reached statistical significance (*p* = 8.088 × 10^−8^, Figure 3).

#### 3.2.3. Apolipoproteins, Phospholipids, and Glycerolipids

Through the application of the IVW analysis method, we found that ApoB (*p* = 5.609 × 10^−12^, Figure 3) and TG (*p* = 1.006 × 10^−5^, Figure 3) were significantly associated with TL. Specifically, for ApoB, each one standard deviation increase was associated with an OR of 1.040 (95% CI: 1.029–1.052) for TL. Each increase of one standard deviation for TG was associated with an OR of 1.029 (95% CI: 1.016–1.042) for TL.

### 3.3. Multivariate MR Analysis Results

In the multivariate MR analysis of Model 1, which assessed the association between *n*-3 PUFAs and *n*-6 PUFAs and TL, we used the multivariable IVW method. The results indicated a positive correlation between *n*-6 PUFAs and TL (OR: 1.037, 95% CI: 1.015–1.059, *p* = 0.001, Table S5). However, the mvMR-Egger intercept results suggested the presence of pleiotropy (*p* = 0.020), and in conjunction with the univariate MR results for fatty acids, there was no significant causal relationship between PUFAs and TL. Therefore, we consider the causal relationship between *n*-6 PUFAs and TL to be potentially unstable. No significant correlation was observed between *n*-3 PUFAs and TL (*p* = 0.128).

In Model 2, the results showed that LDL-C, VLDL-C, ApoB, TG, and total cholesterol were not significantly correlated with TL (Table S5). For the other exposures, no significant correlations were found either. The results for Models 3 and 4 also indicated no significant correlation between HDL-C and ApoA1 and TL.

## 4. Discussion

Our study utilized comprehensive MR methods, including uvMR and mvMR, to analyze the causal effects between 21 lipids, apolipoproteins, and TL. By combining the results of univariate and multivariate MR, we found that MUFA, the MUFA/FA ratio, LDL-C, VLDL-C, total cholesterol, ApoB, and TG may have suggestive positive causal relationships with TL. According to MR findings, we can deduce discrepancies in the causal associations between this study’s five major categories of exposure factors and TL. Phospholipids and glycerides do not manifest a statistically significant causal link with TL. However, fatty acids, apolipoproteins, lipoproteins, and cholesterol exhibit a multifaceted pattern of causality with TL rather than a uniform causal model. Hence, our study concludes that categorizing fatty acids alone is insufficient for establishing their causal relationship with TL.

To date, despite earlier research into the relationship between lipids, apolipoproteins, and TL, epidemiological and clinical studies have not reached conclusive findings, and relevant research remains relatively limited. Regarding the mechanisms of action between lipids and TL, despite discussions, the specific mechanisms are far from clear. Possible mechanisms involve multiple aspects. Firstly, lipid and fatty acid metabolism may affect TL through oxidative stress. Oxidative stress is considered one of the factors contributing to aging [32,33,34], yet it may also slow telomere attrition [35,36]. Previous studies have suggested that the accumulation of fat in the body may be related to oxidative stress [37], and oxidative stress plays a role in the development of age-related diseases such as metabolic syndrome [37,38]. Metabolic syndrome, in turn, is associated with oxidative damage to DNA and lipid levels as well as telomere shortening [38]. Additionally, lipid metabolism is closely related to the inflammatory process, and inflammation is one factor affecting TL in the bloodstream [39,40,41]. It’s worth noting that there exists a complex interplay between oxidative stress and inflammation [33,39].

The relationship between *n*-3 PUFAs and TL has been contradictory in epidemiological and clinical studies. As an essential dietary component, *n*-3 PUFAs, due to their unique biochemical properties, may influence telomere biology. The study by Farzaneh-Far et al. [42] has laid a critical foundation for our understanding of the impact of *n*-3 PUFAs on TL. They conducted a prospective study involving 608 patients with stable coronary artery disease. The study results demonstrated a negative correlation between baseline blood levels of *n*-3 PUFAs (including DHA and EPA) and the rate of telomere shortening over five years (OR, 0.68; 95% CI, 0.47–0.98). In another cross-sectional study measuring leukocyte TL in 2284 women through survey questionnaires, it was found that while the total fat intake was unrelated to TL, the intake of PUFAs, particularly linoleic acid, was negatively correlated with TL [43]. However, in a separate cross-sectional study, the authors conducted a randomized controlled trial involving 344 participants and found no significant correlation between dietary PUFAs and TL [44]. Furthermore, additional research findings suggest a positive correlation between *n*-3 PUFAs levels and TL. In the study, Chang et al. [20] conducted a case–control study of patients with coronary artery disease. They used linear regression analysis to assess the relationship between plasma PUFAs and genetic variations. The study concluded that a higher *n*-6/*n*-3 PUFAs ratio in plasma and lower levels of EPA and DHA were positively associated with shorter TL in the Chinese population. In a study encompassing 46 obese children aged 3–4 years [45], researchers measured leukocyte telomere length and employed gas chromatography to determine the levels of six fatty acids in red blood cells, including SFAs, *n*-3 PUFAs, *n*-6 PUFAs, arachidonic acid (AA), and DHA. Their study results indicated that a reduction in DHA content and an increase in the AA/DHA ratio may be associated with telomere shortening. Furthermore, in another randomized double-blind controlled trial [46], researchers recruited 85 participants aged 25 to 75 with chronic kidney function impairment and categorized them into different groups based on their dietary habits. The study found that, among patients with chronic kidney function impairment, those who supplemented with *n*-3 PUFAs exhibited an increase in neutrophil TL compared to other groups.

Several factors may contribute to the differences in these study results. Firstly, there is a wide variety of methods for measuring fatty acid levels, including gas chromatography, mass spectrometry, and food frequency questionnaires, and different measurement methods can lead to differences in results. Additionally, some studies have small sample sizes or lack long-term dietary information, making it difficult to adequately reflect changes in *n*-3 PUFA levels in study participants over many years, which is crucial for telomere biology research. Our study found no significant correlation between *n*-3 PUFAs and TL, consistent with related research conclusions.

In our study, no causal relationship was found between *n*-6 PUFAs and TL. One study’s findings indicated that the intake of total *n*-6 PUFAs, which includes 98.9% linoleic acid (LA), was unrelated to TL [47]. Additionally, research has shown that LA intake does not correlate with any inflammatory markers [48]. Finally, few studies have demonstrated the adverse effects of *n*-6 PUFAs or their association with the risk of chronic diseases [49]. These studies may contribute to the explanation of our conclusions. It is worth noting that this study suggests a potential association between *n*-6 PUFAs and TL; however, our multivariable MR results indicate no significant correlation between PUFA, PUFA/FA, and TL, and there is a pleiotropy issue in the multi-instrument MR results for *n*-6 PUFAs. Therefore, collectively, this study suggests that both *n*-3 PUFAs and *n*-6 PUFAs may not have significant correlations with TL.

The research on the correlation between lipoproteins and TL is limited and has yielded inconsistent conclusions. One study, involving 4944 participants, found an association between TL and the oldest age group’s LDL-C levels, as measured by real-time quantitative polymerase chain reaction. However, no correlation was observed with TG or HDL-C [50]. In contrast, another study based on the NHANES database showed no association between TL and LDL-C or TG but demonstrated a positive correlation with HDL-C when TL was less than 1.25 [51]. Notably, Nawrot et al.’s research indicated a link between higher levels of oxidized low-density lipoprotein and shorter leukocyte telomeres [52]. Furthermore, a study involving 82 healthy subjects found a negative correlation between TL and TG and a positive correlation with HDL-C [53]. Aulinas et al. [54] investigated the relationship between TL and adipokine balance in 154 patients with Cushing’s syndrome. Their study showed a negative correlation between total cholesterol, TG, and TL. In another study examining the relationship between TL and inflammation in 83 elderly women aged 65 to 74, HDL-C, LDL-C, and TG were found to be unrelated to TL [55]. Similarly, Katarina et al. found no association between TL and cholesterol, serum LDL-C, or serum TG [56]. However, the relationship between TG and TL still lacks definitive conclusions. Our study suggests that there may be a potential positive causal relationship between TG and TL, although further research is needed to substantiate this evidence.

Lee et al. [57] conducted a cross-sectional study involving 309 participants aged between 8 and 80 years, measuring average TL and examining BMI and cardiovascular risk factors. Their study concluded a negative correlation between TL and ApoB. Research on the relationship between ApoB and TL is relatively limited; however, our study’s results suggest that there may be a potential positive causal relationship between ApoB and TL. This conclusion provides intriguing avenues for future research to further elucidate this association’s mechanisms and biological significance.

In summary, consensus on the relationship between lipids and TL has not yet been reached. However, our study suggests potential positive causal relationships between LDL-C, VLDL-C, total cholesterol, ApoB, TG, and TL. The erosion of telomeres is closely associated with the aging process and the increased risk of age-related diseases. Consequently, there is widespread interest and emphasis on the potential of lipid supplementation to slow telomere attrition. This study further substantiates the beneficial role of lipids and lipoproteins in TL. This finding could heighten clinical attention to lipids and contribute to the prevention of aging and associated diseases. Patients at risk may potentially benefit from preventive lipid supplementation.

Our study has several strengths. Firstly, we used MR to estimate causal relationships, effectively mitigating the impact of confounding bias compared to traditional observational studies and addressing the challenge of establishing causality in previous cross-sectional research. Secondly, we employed comprehensive GWAS data for MR analysis to enhance the accuracy of our estimates. Genetic information is determined before any confounding clinical factors come into play, reducing their influence. Thirdly, to ensure result accuracy, we applied various statistical tools, such as using the F-statistic to exclude the influence of weak instrumental variables and utilizing the MR-Presso method to identify potential outliers. Additionally, we conducted heterogeneity and multiplicity analyses to ensure the reliability of MR results.

However, our study also has limitations. Firstly, our data is limited to individuals of European ancestry, so caution should be exercised when extrapolating these findings to populations of different races. Secondly, despite conducting various sensitivity analyses to test MR assumptions, we cannot completely rule out the influence of confounding bias and/or horizontal pleiotropy. Lastly, as we used genetic data, we can only infer causal relationships between exposure and outcome without specifying the exact mechanisms involved.

## 5. Conclusions

Our study, utilizing genetic data, provides preliminary evidence supporting potential positive causal relationships between MUFA, MUFA/FA, LDL-C, VLDL-C, total cholesterol, ApoB, TG, and TL.

## Data Availability

Data sharing is not applicable to this article.

## Supplementary Materials

The following supporting information can be downloaded at: https://www.mdpi.com/article/10.3390/nu15214497/s1, Table S1: Information of eQTL and GWAS summary data. Table S2: Genetic variants used as instrumental variables in univariable MR and the corresponding F statistics. Table S3: Cochran Q test result and MR-Egger intercept in univariable MR. Table S4: MR-PRESSO analysis for the association between lipids, apolipoproteins and telomere length. Table S5: Causal effects of lipid-related traits levels on sepsis via multivariable IVW MR analyses.
